# 2-(2-Furylmethyl­amino­meth­yl)-4-sulfanylphenol

**DOI:** 10.1107/S1600536809032401

**Published:** 2009-08-22

**Authors:** Wu Chen, Lian Liu, Di Xu, Qing-Fu Zeng

**Affiliations:** a Engineering Research Center for Clean Production of Textile Dyeing and Printing, Ministry of Education, Wuhan 430073, People’s Republic of China

## Abstract

In the title compound, C_12_H_13_NO_2_S, the dihedral angle between the furan and benzene rings is 62.2 (2)° and an intra­molecular O—H⋯N hydrogen bond is formed. In the crystal, mol­ecules are linked by weak inter­molecular N—H⋯S hydrogen bonds.

## Related literature

For background, see: Shi *et al.* (2007 ▶). For reference structural data, see: Allen *et al.* (1987 ▶).
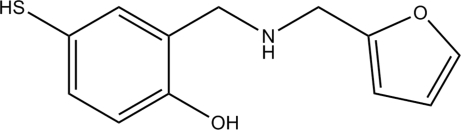


## Experimental

### 

#### Crystal data

C_12_H_13_NO_2_S
*M* *_r_* = 235.29
Orthorhombic, 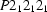

*a* = 5.5778 (12) Å
*b* = 13.589 (3) Å
*c* = 14.943 (3) Å
*V* = 1132.6 (4) Å^3^
*Z* = 4
Mo *K*α radiation
μ = 0.27 mm^−1^
*T* = 293 K
0.30 × 0.30 × 0.10 mm

#### Data collection

Enraf–Nonius CAD4 diffractometer
Absorption correction: ψ scan (North *et al.*, 1968 ▶) *T*_min_ = 0.924, *T*_max_ = 0.974
2528 measured reflections
2216 independent reflections
1811 reflections with *I* > 2σ(*I*)
*R*_int_ = 0.034
3 standard reflections every 200 reflections intensity decay: 1%

#### Refinement

*R*[*F* ^2^ > 2σ(*F* ^2^)] = 0.058
*wR*(*F* ^2^) = 0.166
*S* = 1.06
2216 reflections
150 parameters
H atoms treated by a mixture of independent and constrained refinement
Δρ_max_ = 0.35 e Å^−3^
Δρ_min_ = −0.46 e Å^−3^
Absolute structure: Flack (1983 ▶), 900 Friedel pairs
Flack parameter: 0.00 (17)

### 

Data collection: *CAD-4 Software* (Enraf–Nonius, 1989 ▶); cell refinement: *CAD-4 Software*; data reduction: *XCAD4* (Harms & Wocadlo, 1995 ▶); program(s) used to solve structure: *SHELXS97* (Sheldrick, 2008 ▶); program(s) used to refine structure: *SHELXL97* (Sheldrick, 2008 ▶); molecular graphics: *SHELXTL* (Sheldrick, 2008 ▶); software used to prepare material for publication: *SHELXTL*.

## Supplementary Material

Crystal structure: contains datablocks global, I. DOI: 10.1107/S1600536809032401/hb5047sup1.cif

Structure factors: contains datablocks I. DOI: 10.1107/S1600536809032401/hb5047Isup2.hkl

Additional supplementary materials:  crystallographic information; 3D view; checkCIF report

## Figures and Tables

**Table 1:** Hydrogen-bond geometry (Å, °)

*D*—H⋯*A*	*D*—H	H⋯*A*	*D*⋯*A*	*D*—H⋯*A*
O2—H2*A*⋯N1	0.82	2.04	2.692 (5)	136
N1—H1*C*⋯S1^i^	0.93 (5)	2.90 (4)	3.605 (3)	134 (3)

Symmetry code: (i) 
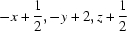
.

**Fig. 1.**
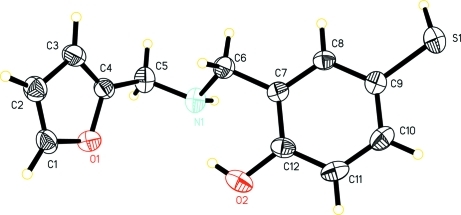
The molecular structure of (I) showing 30% probability displacement ellipsoids.

## supplementary crystallographic information

### Comment

There has been much research interest in Schiff base compounds due to their biological activities (Shi *et al.*, 2007). In this work, we report here the crystal structure of the title compound, (I). In (I), all bond lengths are within normal ranges (Allen *et al.*, 1987) (Fig. 1). There are an intramolecular O-H···N hydrogen bond and an intermolecular N-H···S hydrogen bond in (I).

### Experimental

A mixture of 2-hydroxy-5-mercaptobenzaldehyde (154 mg, 1 mmol) and furan-2-ylmethanamine (97 mg, 1 mmol) were stirred in methanol (10 ml) for 2 h. Then NaBH_4_ (76 mg, 2 mmol) was added to the reaction solution slowly, and stirred at room temperature for 2 h. The mixture was evaporated under vacuum, and dissolved in dichloromethane (5 ml). The solution was washed with saturated NaCl solution and water, respectively, dried over anhydrous sodium sulfate, and evaporated. Purification by silica gel afforded pure product. Colourless blocks of (I) were obtained by recrystallization of the pure product in methanol.

### Refinement

The N-bound H atom was located in a difference map and its position was freely refined. The other H atoms were positioned geometrically (C—H = 0.93–0.97Å, O—H = 0.82Å, S—H = 1.20Å) and refined as riding, with *U*_iso_(H) = 1.2*U*_eq_(carrier) or 1.5*U*_eq_(methyl C).

### Crystal data

**Table:**

C_12_H_13_NO_2_S	*F*(000) = 496
*M**_r_* = 235.29	*D*_x_ = 1.380 Mg m^−^^3^
Orthorhombic, *P*2_1_2_1_2_1_	Mo *K*α radiation, λ = 0.71073 Å
Hall symbol: P 2ac 2ab	Cell parameters from 25 reflections
*a* = 5.5778 (12) Å	θ = 9–12°
*b* = 13.589 (3) Å	µ = 0.27 mm^−^^1^
*c* = 14.943 (3) Å	*T* = 293 K
*V* = 1132.6 (4) Å^3^	Block, colorless
*Z* = 4	0.30 × 0.30 × 0.10 mm

### Data collection

**Table:**

Enraf–Nonius CAD4 diffractometer	1811 reflections with *I* > 2σ(*I*)
Radiation source: fine-focus sealed tube	*R*_int_ = 0.034
graphite	θ_max_ = 26.0°, θ_min_ = 2.0°
ω/2θ scans	*h* = −6→0
Absorption correction: ψ scan (North *et al.*, 1968)	*k* = −16→16
*T*_min_ = 0.924, *T*_max_ = 0.974	*l* = −18→0
2528 measured reflections	3 standard reflections every 200 reflections
2216 independent reflections	intensity decay: 1%

### Refinement

**Table:**

Refinement on *F*^2^	Hydrogen site location: inferred from neighbouring sites
Least-squares matrix: full	H atoms treated by a mixture of independent and constrained refinement
*R*[*F*^2^ > 2σ(*F*^2^)] = 0.058	*w* = 1/[σ^2^(*F*_o_^2^) + (0.1031*P*)^2^ + 0.1612*P*] where *P* = (*F*_o_^2^ + 2*F*_c_^2^)/3
*wR*(*F*^2^) = 0.166	(Δ/σ)_max_ = 0.002
*S* = 1.06	Δρ_max_ = 0.35 e Å^−^^3^
2216 reflections	Δρ_min_ = −0.46 e Å^−^^3^
150 parameters	Extinction correction: *SHELXL97* (Sheldrick, 2008), Fc^*^=kFc[1+0.001xFc^2^λ^3^/sin(2θ)]^-1/4^
0 restraints	Extinction coefficient: 0.058 (9)
Primary atom site location: structure-invariant direct methods	Absolute structure: Flack (1983), 900 Friedel pairs
Secondary atom site location: difference Fourier map	Flack parameter: 0.00 (17)

### Special details

**Table:**

Geometry. All e.s.d.'s (except the e.s.d. in the dihedral angle between two l.s. planes) are estimated using the full covariance matrix. The cell e.s.d.'s are taken into account individually in the estimation of e.s.d.'s in distances, angles and torsion angles; correlations between e.s.d.'s in cell parameters are only used when they are defined by crystal symmetry. An approximate (isotropic) treatment of cell e.s.d.'s is used for estimating e.s.d.'s involving l.s. planes.
Refinement. Refinement of *F*^2^ against ALL reflections. The weighted *R*-factor *wR* and goodness of fit *S* are based on *F*^2^, conventional *R*-factors *R* are based on *F*, with *F* set to zero for negative *F*^2^. The threshold expression of *F*^2^ > σ(*F*^2^) is used only for calculating *R*-factors(gt) *etc*. and is not relevant to the choice of reflections for refinement. *R*-factors based on *F*^2^ are statistically about twice as large as those based on *F*, and *R*- factors based on ALL data will be even larger.

### Fractional atomic coordinates and isotropic or equivalent isotropic displacement parameters (Å^2^)

**Table:**

	*x*	*y*	*z*	*U*_iso_*/*U*_eq_	
C1	−0.2480 (7)	0.7259 (3)	0.4206 (3)	0.0591 (10)	
H1	−0.3948	0.7288	0.4501	0.071*	
C2	−0.1233 (8)	0.6441 (3)	0.4068 (3)	0.0581 (9)	
H2	−0.1659	0.5807	0.4239	0.070*	
C3	0.0881 (8)	0.6728 (3)	0.3607 (3)	0.0564 (10)	
H3	0.2108	0.6312	0.3420	0.068*	
C4	0.0791 (6)	0.7696 (3)	0.3490 (2)	0.0480 (8)	
C5	0.2431 (8)	0.8424 (3)	0.3079 (3)	0.0600 (10)	
H5A	0.2795	0.8933	0.3512	0.072*	
H5B	0.3923	0.8100	0.2920	0.072*	
C6	0.1385 (8)	0.8197 (2)	0.1507 (2)	0.0526 (9)	
H6A	0.0551	0.7597	0.1671	0.063*	
H6B	0.3023	0.8025	0.1354	0.063*	
C7	0.0183 (6)	0.8652 (2)	0.0710 (2)	0.0422 (8)	
C8	0.1130 (6)	0.8552 (2)	−0.0141 (2)	0.0449 (8)	
H8	0.2558	0.8209	−0.0219	0.054*	
C9	−0.0009 (7)	0.8953 (3)	−0.0874 (3)	0.0481 (8)	
C10	−0.2099 (8)	0.9468 (3)	−0.0782 (3)	0.0591 (10)	
H10	−0.2856	0.9737	−0.1280	0.071*	
C11	−0.3069 (7)	0.9583 (3)	0.0062 (3)	0.0613 (11)	
H11	−0.4480	0.9939	0.0134	0.074*	
C12	−0.1958 (6)	0.9172 (3)	0.0804 (3)	0.0502 (9)	
H1C	0.230 (9)	0.942 (3)	0.212 (3)	0.060*	
N1	0.1391 (7)	0.8878 (2)	0.2275 (2)	0.0550 (8)	
O1	−0.1310 (6)	0.8050 (2)	0.38570 (18)	0.0632 (8)	
O2	−0.2984 (5)	0.9310 (2)	0.1623 (2)	0.0696 (8)	
H2A	−0.2170	0.9038	0.2007	0.104*	
S1	0.1282 (2)	0.88051 (8)	−0.19340 (6)	0.0635 (4)	
H1A	0.3423	0.8770	−0.1863	0.095*	

### Atomic displacement parameters (Å^2^)

**Table:**

	*U*^11^	*U*^22^	*U*^33^	*U*^12^	*U*^13^	*U*^23^
C1	0.041 (2)	0.091 (3)	0.0460 (19)	0.001 (2)	0.0025 (17)	0.011 (2)
C2	0.052 (2)	0.063 (2)	0.059 (2)	−0.006 (2)	0.005 (2)	0.0063 (17)
C3	0.053 (2)	0.058 (2)	0.057 (2)	0.0042 (18)	0.010 (2)	−0.0024 (18)
C4	0.0402 (19)	0.062 (2)	0.0419 (17)	0.0025 (16)	−0.0010 (15)	−0.0007 (16)
C5	0.058 (2)	0.068 (2)	0.054 (2)	−0.015 (2)	−0.007 (2)	0.0067 (19)
C6	0.055 (2)	0.0490 (18)	0.054 (2)	0.0003 (19)	0.002 (2)	0.0033 (16)
C7	0.0361 (17)	0.0396 (16)	0.0508 (19)	−0.0041 (15)	−0.0016 (15)	−0.0001 (14)
C8	0.0382 (17)	0.0426 (16)	0.0540 (19)	0.0009 (15)	0.0022 (17)	−0.0009 (14)
C9	0.047 (2)	0.0470 (18)	0.0501 (19)	−0.0058 (17)	−0.0034 (17)	0.0017 (15)
C10	0.054 (2)	0.057 (2)	0.066 (2)	0.0046 (19)	−0.017 (2)	0.0031 (19)
C11	0.041 (2)	0.060 (2)	0.083 (3)	0.0110 (17)	−0.007 (2)	−0.002 (2)
C12	0.0385 (19)	0.0486 (18)	0.064 (2)	−0.0037 (15)	0.0074 (17)	−0.0040 (17)
N1	0.064 (2)	0.0487 (16)	0.0518 (16)	−0.0126 (18)	−0.0062 (16)	0.0024 (14)
O1	0.0598 (17)	0.0678 (16)	0.0619 (16)	0.0180 (15)	0.0025 (15)	0.0038 (12)
O2	0.0543 (17)	0.0815 (18)	0.0731 (19)	0.0024 (15)	0.0224 (15)	−0.0063 (15)
S1	0.0710 (7)	0.0711 (7)	0.0486 (5)	−0.0030 (6)	0.0064 (5)	0.0029 (4)

### Geometric parameters (Å, °)

**Table:**

C1—C2	1.328 (6)	C6—H6B	0.9700
C1—O1	1.361 (5)	C7—C8	1.383 (5)
C1—H1	0.9300	C7—C12	1.394 (5)
C2—C3	1.420 (6)	C8—C9	1.379 (5)
C2—H2	0.9300	C8—H8	0.9300
C3—C4	1.327 (5)	C9—C10	1.366 (6)
C3—H3	0.9300	C9—S1	1.752 (4)
C4—O1	1.381 (5)	C10—C11	1.382 (6)
C4—C5	1.481 (5)	C10—H10	0.9300
C5—N1	1.470 (5)	C11—C12	1.388 (6)
C5—H5A	0.9700	C11—H11	0.9300
C5—H5B	0.9700	C12—O2	1.365 (5)
C6—N1	1.473 (5)	N1—H1C	0.93 (5)
C6—C7	1.501 (5)	O2—H2A	0.8200
C6—H6A	0.9700	S1—H1A	1.2000
			
C2—C1—O1	110.5 (3)	C8—C7—C12	118.0 (3)
C2—C1—H1	124.8	C8—C7—C6	121.2 (3)
O1—C1—H1	124.8	C12—C7—C6	120.7 (3)
C1—C2—C3	106.3 (4)	C9—C8—C7	121.0 (3)
C1—C2—H2	126.8	C9—C8—H8	119.5
C3—C2—H2	126.8	C7—C8—H8	119.5
C4—C3—C2	107.7 (4)	C10—C9—C8	121.1 (4)
C4—C3—H3	126.1	C10—C9—S1	120.0 (3)
C2—C3—H3	126.1	C8—C9—S1	118.9 (3)
C3—C4—O1	108.9 (3)	C9—C10—C11	118.9 (4)
C3—C4—C5	133.9 (4)	C9—C10—H10	120.6
O1—C4—C5	117.2 (3)	C11—C10—H10	120.6
N1—C5—C4	112.1 (3)	C10—C11—C12	120.6 (4)
N1—C5—H5A	109.2	C10—C11—H11	119.7
C4—C5—H5A	109.2	C12—C11—H11	119.7
N1—C5—H5B	109.2	O2—C12—C11	118.3 (3)
C4—C5—H5B	109.2	O2—C12—C7	121.3 (4)
H5A—C5—H5B	107.9	C11—C12—C7	120.4 (4)
N1—C6—C7	111.1 (3)	C5—N1—C6	111.9 (3)
N1—C6—H6A	109.4	C5—N1—H1C	109 (3)
C7—C6—H6A	109.4	C6—N1—H1C	108 (2)
N1—C6—H6B	109.4	C1—O1—C4	106.5 (3)
C7—C6—H6B	109.4	C12—O2—H2A	109.5
H6A—C6—H6B	108.0	C9—S1—H1A	109.5
			
O1—C1—C2—C3	0.5 (5)	S1—C9—C10—C11	−179.4 (3)
C1—C2—C3—C4	−0.2 (5)	C9—C10—C11—C12	−0.8 (6)
C2—C3—C4—O1	−0.1 (4)	C10—C11—C12—O2	179.7 (4)
C2—C3—C4—C5	178.8 (4)	C10—C11—C12—C7	1.3 (6)
C3—C4—C5—N1	114.6 (5)	C8—C7—C12—O2	−179.2 (3)
O1—C4—C5—N1	−66.5 (4)	C6—C7—C12—O2	2.2 (5)
N1—C6—C7—C8	137.6 (3)	C8—C7—C12—C11	−0.8 (5)
N1—C6—C7—C12	−43.8 (5)	C6—C7—C12—C11	−179.4 (3)
C12—C7—C8—C9	−0.1 (5)	C4—C5—N1—C6	−73.9 (4)
C6—C7—C8—C9	178.6 (3)	C7—C6—N1—C5	176.6 (3)
C7—C8—C9—C10	0.5 (5)	C2—C1—O1—C4	−0.6 (4)
C7—C8—C9—S1	179.8 (3)	C3—C4—O1—C1	0.4 (4)
C8—C9—C10—C11	0.0 (6)	C5—C4—O1—C1	−178.7 (3)

### Hydrogen-bond geometry (Å, °)

**Table:**

*D*—H···*A*	*D*—H	H···*A*	*D*···*A*	*D*—H···*A*
O2—H2A···N1	0.82	2.04	2.692 (5)	136
N1—H1C···S1^i^	0.93 (5)	2.90 (4)	3.605 (3)	134 (3)

Symmetry codes: (i) −*x*+1/2, −*y*+2, *z*+1/2.
